# Children’s route choice during active transportation to school: difference between shortest and actual route

**DOI:** 10.1186/s12966-016-0373-y

**Published:** 2016-04-12

**Authors:** Dirk Dessing, Sanne I. de Vries, Geertje Hegeman, Evert Verhagen, Willem van Mechelen, Frank H. Pierik

**Affiliations:** Department of Public & Occupational Health and EMGO+ Institute, VU University Medical Center, Amsterdam, The Netherlands; Amsterdam Collaboration on Health & Safety in Sports, IOC Research Center, AMC/VUmc, Amsterdam, The Netherlands; TNO, Department of Life Style, Leiden, The Netherlands; Research group Healthy Lifestyle in a Supporting Environment, The Hague University of Applied Sciences, The Hague, The Netherlands; Royal HaskoningDHV, Amersfoort, The Netherlands; Australian Centre for Research into Injury in Sport and its Prevention (ACRISP), Federation University Australia, Ballarat, Australia; Division of Exercise Science and Sports Medicine (ESSM), Department of Human Biology, Faculty of Health Sciences, University of Cape Town, Cape Town, South Africa; TNO, Department of Urban Environment and Safety, Utrecht, The Netherlands; School of Human Movement and Nutrition Sciences, Faculty of Health and Behavioural Sciences, University of Queensland, Brisbane, Australia; School of Public Health, Physiotherapy and Population Sciences, University College Dublin, Dublin, Ireland

**Keywords:** Elementary school, Children, Global Positioning System (GPS), Built environment, GIS, Active transportation, Walking, Cycling

## Abstract

**Background:**

The purpose of this study is to increase our understanding of environmental correlates that are associated with route choice during active transportation to school (ATS) by comparing characteristics of actual walking and cycling routes between home and school with the shortest possible route to school.

**Methods:**

Children (*n* = 184; 86 boys, 98 girls; age range: 8–12 years) from seven schools in suburban municipalities in the Netherlands participated in the study. Actual walking and cycling routes to school were measured with a GPS-device that children wore during an entire school week. Measurements were conducted in the period April–June 2014. Route characteristics for both actual and shortest routes between home and school were determined for a buffer of 25 m from the routes and divided into four categories: Land use (residential, commercial, recreational, traffic areas), Aesthetics (presence of greenery/natural water ways along route), Traffic (safety measures such as traffic lights, zebra crossings, speed bumps) and Type of street (pedestrian, cycling, residential streets, arterial roads). Comparison of characteristics of shortest and actual routes was performed with conditional logistic regression models.

**Results:**

Median distance of the actual walking routes was 390.1 m, whereas median distance of actual cycling routes was 673.9 m. Actual walking and cycling routes were not significantly longer than the shortest possible routes. Children mainly traveled through residential areas on their way to school (>80 % of the route). Traffic lights were found to be positively associated with route choice during ATS. Zebra crossings were less often present along the actual routes (walking: OR = 0.17, 95 % CI = 0.05–0.58; cycling: OR = 0.31, 95 % CI = 0.14–0.67), and streets with a high occurrence of accidents were less often used during cycling to school (OR = 0.57, 95 % CI = 0.43–0.76). Moreover, percentage of visible surface water along the actual route was higher compared to the shortest routes (walking: OR = 1.04, 95 % CI = 1.01–1.07; cycling: OR = 1.03, 95 % CI = 1.01–1.05).

**Discussion:**

This study showed a novel approach to examine built environmental exposure during active transport to school. Most of the results of the study suggest that children avoid to walk or cycle along busy roads on their way to school.

**Electronic supplementary material:**

The online version of this article (doi:10.1186/s12966-016-0373-y) contains supplementary material, which is available to authorized users.

## Background

Stimulating children to be physically active is an important public health promotion and disease prevention strategy [1, 2]. The majority of children in the Netherlands currently do not comply with the latest physical activity guidelines [3, 4]. A promising way to increase children’s habitual daily physical activity is to stimulate active transportation (i.e., walking or cycling) to and from school [5, 6]. Children who use active transportation to school have higher overall levels of physical activity compared to children who rely on motorized transport [7, 8]. Moreover, engaging in active transport is associated with increased levels of physical fitness in children [9].

To further promote physical activity in primary school children, determinants associated with active transportation need to be investigated. Socio-ecological frameworks suggest that certain characteristics of the built environment are important for stimulating active transportation [10, 11]. However, consistent evidence for such associations appears to be lacking. The only environmental characteristic that has been consistently found to be negatively associated with children’s active transportation is the distance between home and school [12]. Most of the literature on determinants of active transportation has focused on environmental characteristics that are related to the transportation mode, i.e., active versus motorized transportation. Another approach to further examine which environmental characteristics stimulate active transportation, is to look at the routes that are used for active transportation. For instance, by comparing characteristics of the actually traveled route with characteristics of the shortest route: exposure to environmental characteristics during actually traveled routes can be significantly different from GIS-modeled routes [13].

Recently, Krenn et al. [14] have performed such an analysis among an adult population of cyclists in Austria. Within this population, environmental characteristics that were associated with the actual cycled route included the presence of bicycle paths, traffic lights, water and greenery, and the absence of dangerous intersections, busy roads, shops, and inclination of the route. Whether the environmental characteristics related to the route choice of adult cyclists also affect the route choice of school children has not been studied yet. It is also unknown to what extent there is a difference between environmental characteristics that are associated with walking routes versus cycling routes to school, but it is likely that different correlates are relevant for walking than for cycling [15].

Apart from a focus on transportation mode rather than route choice, and the lack of discrimination between walking and cycling, another limitation of previous studies is the methodology. This might explain the lack of consistency in observed associations between the built environment and active transportation. Methodological issues include: 1) inaccurate geocoding of the home and school address, 2) different ways to measure the environment, i.e., buffer method, 3) inaccurate estimation of the route to school, and 4) poor quality data of the pedestrian street network [12]. Some of these issues can be addressed by using Global Positioning Systems (GPS) to map the school journey [16].

Firstly, most studies that examined active transportation to school used postal codes for geocoding the location of the home and school address. This method leads to misrepresentations of the environment that children are exposed to. For example, Bow showed that >10 % of the addresses they geocoded based on postal code were further than 200 m from the true address location [17]. With GPS the home address can be determined more accurately by clustering of data points collected during the night, when children are at home [18].

Secondly, in a traditional circular neighborhood buffer approach [19], in which a circle is drawn around the home and school address to assess the home/school environment within a certain range, it is arbitrary what buffer size best represents the environment of the children. Changes in buffer size, e.g., using a 400 m buffer instead of 800 m buffer, can result in very different determinants. A small buffer along the GPS route gives a more precise indication of the actual exposure to the built environment, compared to large circular buffers around the home or school [20, 21].

Third, previous studies have mostly used a Geographic Information Systems (GIS) derived path on the network to represent the actual route traveled. Duncan and others [22] have shown that these GIS derived routes are very similar in distance, but not always representative of the actually traveled route, because different routes were used. Thus, with the use of GPS, several methodological constraints of previous studies can be avoided and characteristics of the actually travelled route can be mapped more precisely [8, 23].

Fourth, in previous studies poor quality data of the pedestrian street network made it difficult to accurately calculate certain characteristics that are believed to be relevant for active transportation, such as network connectivity [24]. Fortunately, a more detailed pedestrian network is increasingly available through satellite imagery and open source websites such as Open Street Map (OSM).

In sum, the aim of this study is to gain a better understanding of the built environmental characteristics that are associated with route choice in both walking and cycling to school as measured by GPS. This study tries to clarify which characteristics of the built environment are associated with children’s active transport to school. The results of this study can support public health professionals and urban planners to create more effective environmental interventions to promote active transportation to school among children.

## Methods

### Participants and setting

This cross-sectional study was conducted in a convenience sample of seven schools participating in the Schoolzone project. The Schoolzone project is a natural experiment in the Netherlands that investigates the effect of increasing traffic safety around primary schools on daily physical activity levels of schoolchildren (ZonMW, project number 525001001). The schools participating in the current study were located in three suburban municipalities in the Amsterdam region: i.e., Zaanstad (*n* = 3), Haarlemmermeer (*n* = 2) and Edam-Volendam (*n* = 2). The neighborhoods in which the seven schools were located were all constructed post-WWII. A total of 342 children attending these schools (grade 6–7, age 8 to 12 years) were invited to participate in the study. Their parents received written information through the school about study goals and procedures. Subsequently, parents provided informed consent for their children. This procedure resulted in a group of 213 (63.8 %) children that were included in the study. The study design and procedures were approved by the medical ethical committee of the VU University Medical Centre, Amsterdam, The Netherlands.

### Instrumentation / Measures

All children were requested to wear a GPS receiver (Travel recorder X, BT-Q1000X, QStarz International Co) during waking hours, for eight consecutive days. The GPS receivers were set to record the geographical position of the children, with a sampling frequency of 5 s. The GPS device was attached to the children’s waist with an elastic belt. The GPS device and belt were handed out during school hours, during which children were personally instructed on how to wear the device. During activities that could damage the device, or could be uncomfortable to wear, the children were asked to temporarily remove the device (e.g., during swimming, showering). Written instructions for children and their parents were handed out together with the device. Furthermore, after receiving the device, children completed a short questionnaire to provide information on their age, gender and habitual daily physical activity behavior. All measurements were conducted between April and June 2014. Out of the 213 children wearing the GPS device, the 184 children that recorded at least one track between home and school were included in the current analysis.

### Data handling

#### a. GPS data

GPS data were downloaded to a computer with Q-Travel v1.48, a travel data management software package from Qstarz. Data was then converted to a csv format for further processing within the URBIS III software package [25]. First, locations of the home address of the children were determined based on clusters in the GPS data recorded during the night time (12 p.m. –6 a.m.) [18]. Location of the school building was determined based on TOP10NL. Next, each GPS track between the home address and the school building was identified with an automatic procedure in URBIS [23], this procedure also includes the tracks that have intermediate stops between home and school, i.e., multi-destination trips. Trips going in both directions (i.e., home or school) were eligible to be included in the analysis. For each track, the calculated speed was used to determine the mode of transport at each individual GPS point. GPS points with a calculated speed below 10 km/h were categorized as ‘walking’. Points were categorized as ‘cycling’ if the speed was between 10 and 25 km/h. The remainder of the GPS points (with a speed below 150 km/h) was categorized as ‘motorized transport’ [23]. To correct for sudden changes in speed due to bad satellite reception, a track was defined as cycling or motorized if there was a period of at least one minute within that transportation mode, i.e., motorized travel, cycling. Tracks that contained both a 1 min period of cycling and a period of motorized travel were categorized as motorized transport. All remaining tracks that were not classified either cycling or motorized were defined as walking tracks. Then, descriptive characteristics, i.e., distance, duration, average speed, and maximum speed were calculated for all recorded tracks. For each child, the shortest actual walking and cycling track were selected to be used in the subsequent analysis of active transportation which compared the actual route with the shortest route via the street network.

#### b. Street Network

The street network was constructed using the road centerlines available in the TOP10NL database (topographic map of the Netherlands, scale 1: 10.000 provided by the Dutch Land use register Kadaster). To complete the network, centerlines of missing streets were manually added by the first author based on Open Street Map (OSM) data and satellite images from LuchtfotoNL (2014). Shortest routes between home and school were generated based on this street network, using the home address, as determined with the above mentioned cluster detection method, and the x and y coordinates of the center of the polygon of the school buildings. Shortest routes were calculated with the Network Analyst tool in ArcGis 10.2. Differences in distance between GIS derived shortest routes and the actual walking and cycling routes were calculated as a detour ratio.

#### c Characteristics of the built environment

**Fig. 1:**
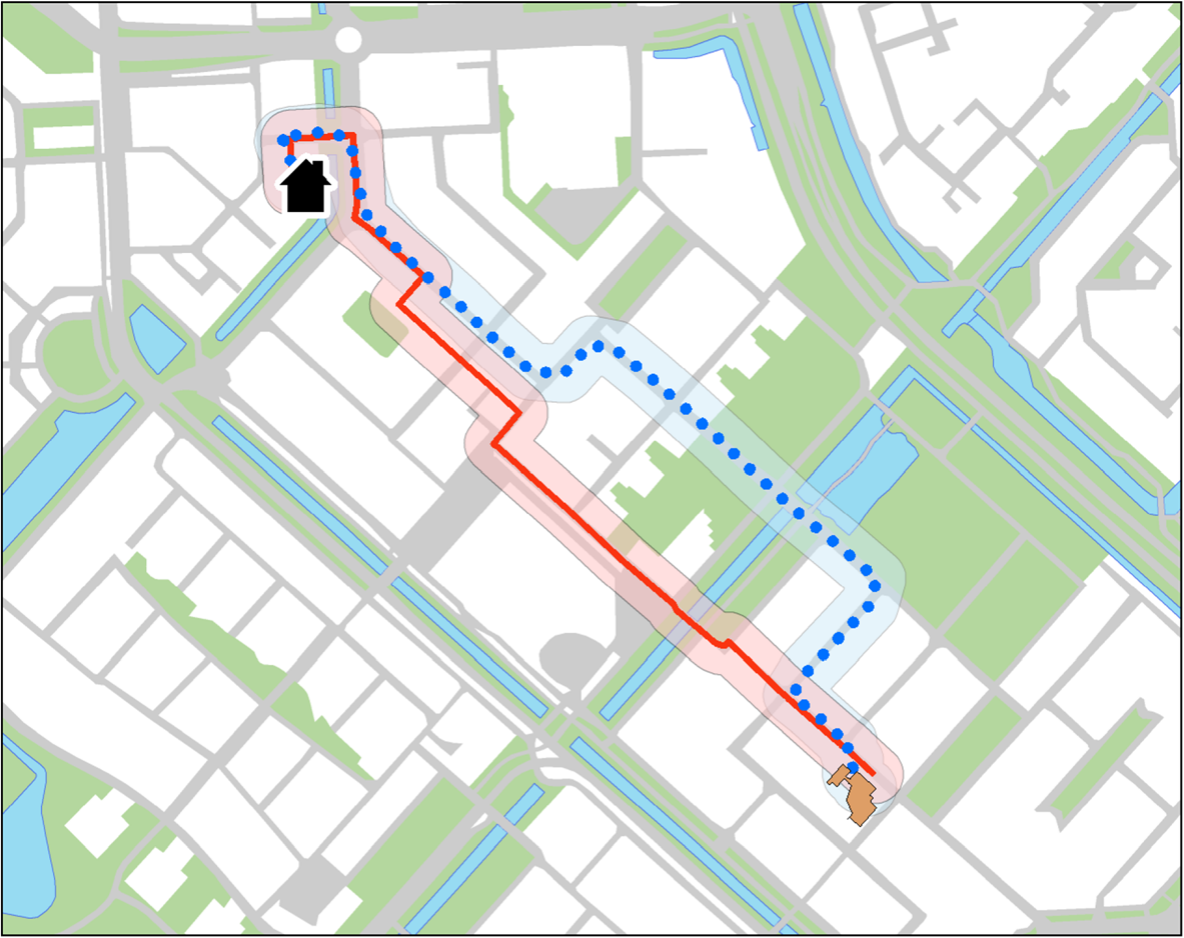
Comparison of shortest route and actual route. Example of a GIS-derived shortest route between home and school on the street network (*red line*) and an actual traveled route as measured with the GPS-device (*dotted blue*). The 25 m buffer was used to measure the environmental characteristics along the routes

For both the shortest and the actual traveled routes, four categories of built environmental characteristics were determined within a buffer distance of 25 m along the routes: land use, traffic, aesthetics, and type of street (see also Fig. 1).

Land use mix was calculated using CBS Land use data, and based on a 4-category entropy index [26]. A distinction was made between the following entropy categories: residential areas, commercial areas, traffic areas, and recreational areas. In this entropy index, 0 stands for no diversity, while 1 means that there is an equal distribution of land use. The number of residents was reported per square kilometer using CBS squares, derived from the Dutch Statistics Center, in 100 by 100 m cells [27]. The residential density was calculated proportionally for the buffer areas that intersected with the cells.

Traffic junctions, traffic accidents, zebra crossings, street lights, traffic lights and speed bumps were all represented by point data. The number of junctions was determined based on the street network from the TOP10NL dataset provided by the Dutch land use registry Kadaster [28]. The number of traffic accidents was derived from BRON, a national database in which traffic related accidents are recorded through official reports or registration sets from the police. Around 84 % of accidents are represented in this database [29]. All other point data were collected using local land use registry data (GBKN) from participating municipalities. The amount of points found within the 25 m buffer along the routes were reported as the number of points per km of route.

The percentage water along the route was determined as a measure of aesthetics of visible surface water (e.g., ponds, rivers, lakes). This percentage was calculated based on polygons of aquatic areas from the TOP10NL database. Similarly, the percentage of green along the route was calculated based on polygons of green from TOP10NL, these represent neighborhood green spaces (e.g., bushes, grass plots, woods). Both percentage of green and water along the route were reported as average percentage per kilometer of route. Number of trees along the route are also reported and are represented by point data.

The type of street was determined based on attributes of the street network from the TOP10NL data. The percentage of street type along the route within a buffer of 25 m was calculated for four street types: residential streets, pedestrian path, separate bicycle path, and arterial roads with a bicycle lane. Residential streets are located in residential areas, but other types of streets are also present in these areas, i.e., cycling paths and pedestrian paths. Separate pedestrian and cycling paths in the Netherlands are usually not accessible to motorized traffic (sometimes with the exception of motor scooters). Residential streets are used by all modes of transportation, thus motorized traffic shares the streets with cyclists while pedestrians are directed to the sidewalk. Maximum speed of motorized traffic on residential streets is usually low, with a maximum of 30 km/h. Arterial roads on the other hand, have a speed limit of 50 km/h. When cyclists travel along these arterial roads, they are directed to a separately marked cycling lane. Pedestrians can use the sidewalk when these are available. A more detailed description of all GIS-variables used in the analysis can be viewed in Additional file 1: Table S1.

### Statistical analysis

Characteristics that showed a non-normal distribution are summarized by their median and interquartile range reported as the 25^th^ percentile and the 75^th^ percentile. Conditional logistic regression analysis was used to examine the difference between environmental characteristics of the actual route and environmental characteristics of the GIS derived shortest route.

Table 1 shows an example of the dataset that was used in the regression analysis. In this table, ID represents the participant, route 0 stands for the shortest route and route 1 for the actual route. Var1 represents the number of trees, Var2 the percentage of sidewalk along the route, etc. First, each environmental characteristic (e.g., Var1) was tested separately using univariate conditional logistic regression. Conditional logistic regression was used because shortest and actual route are not independent within a child.

**Table 1:** Example of dataset used in the conditional regression analysis

ID	Route	var1	var2	var3	var_i_
		Distance	Trees	%Sidewalk	
1	0	623	22	87	…
1	1	519	12	95	…
2	0	432	20	86	…
2	1	316	19	87	…
3	0	1023	50	75	…
3	1	939	32	78	…
ID_i_	…	…	…	…	…

Then, after selection of candidate variables that had a significance level below 0.20, a backward selection process was used to construct a multivariate model both for environmental characteristics of the walking routes, and for environmental characteristics of the cycling routes. All statistical analyses were performed in SPSS version 22.0.

## Results

In total, a group of 184 children (86 boys, 98 girls, age 8–12 years) recorded 1249 GPS tracks between home and school. General characteristics of the final study population are shown in Table 2. Out of all children, 67 recorded one or more walking tracks to school, and 162 children recorded one or more tracks that were classified as cycling. Eight children did not record any walking or cycling tracks between home and school. Characteristics of the shortest GPS tracks used in the comparison with the shortest GIS routes are shown in Table 3. Characteristics of all 1249 GPS tracks are shown in Additional file 1: Table S2. The median distance of the recorded walking routes was 390.0 (interquartile range: 248.8–606.7) meters with a median duration of 6.1 (interquartile range: 3.8–9.3) minutes. Recorded walking routes were on average 5.6 % longer than the shortest walking routes, but this difference was not significant (p = 0.38). The median distance of the actual traveled cycling route was 673.9 (interquartile range:459.4–1008.3) meters, with a median duration of 5.0 (interquartile range: 3.5–7.6) minutes. On average, actual cycling routes were 10.9 %, but not significantly, longer than the shortest routes over the network (p = 0.11). With the current buffer size of 25 m, median overlap between the two buffered routes was 64 % (interquartile range: 33.4–81.7 %) for walking routes, and 69.3 % (interquartile range: 48.8–86.2 %) for cycling routes.

**Table 2:** General characteristics of the final study population

	n	Mean ± SD
Age (years)	184	10.5 ± 0.9
	n	%
Gender		
Boy	86	46.7
Girl	98	53.3
City		
Volendam	63	34.2
School A	38	20.7
School B	25	13.6
Zaandam	63	34.2
School C	28	15.2
School D	14	7.6
School E	21	11.4
Hoofddorp	58	31.5
School F	32	18.2
School G	26	14.1
Journey to school		
Number of children with at least one walking track	67	36.4
Number of children with at least one cycling track	162	88.0
Number of children with at least one motorized track	70	38.0

**Table 3:** Descriptive statistics of the shortest traveled walking and cycling routes of 176 children

Mode of transport		Mean	Standard deviation	Median	25^th^ percentile	75^th^ percentile
**Walking**	Distance (meters)	462.1	360.5	390.1	248.8	606.7
*N* = 67	Duration (minutes)	8.0	7.2	6.1	3.8	9.3
*n* = 67	Average speed (km/h)	3.8	1.2	3.9	3.0	4.6
	Max Speed (km/h)	12.0	12.1	9.4	7.0	11.2
**Cycling**	Distance (meters)	894.4	891.1	673.9	459.4	1008.3
*N* = 162	Duration (minutes)	9.3	19.6	5.0	3.5	7.6
*n* = 162	Average speed (km/h)	8.2	3.6	8.0	5.5	10.6
	Max Speed (km/h)	18.6	8.4	17.8	15.1	20.9

*N* = number of tracks used in the analysis, *n* = number of children

### Environmental characteristics of walking routes

Results of the conditional logistic regression analyses of walking routes are shown in Table 4, in which environmental characteristics are divided into four categories: land use, traffic, aesthetics and type of street. The majority of the actual walking routes to school passed through residential areas (88.9 % of the route). On average, actual walking routes were going through a significantly smaller amount of transport areas (OR = 0.76, 95 % CI = 0.6–0.96) compared to the shortest route. Moreover, there were significantly fewer street lights (OR = 0.97, 95 % CI = 0.95–0.99) and zebra crossings (OR = 0.55, 95 % CI = 0.35–0.87) within the 25 m buffer of the actual walking route, compared to the shortest walking route. The percentage of sidewalks was lower along the actually traveled routes, compared to the shortest routes (OR = 0.94, CI = 0.90–0.98). Furthermore, around half of the walks to school was conducted on residential streets (49.6 %), which is significantly more than on the shortest route (OR = 1.04, 95 % CI = 1.01–1.08). Shortcuts between houses (%pedestrian paths) were used less (OR = 0.93, 95 % CI = 0.89–0.98) on the actual walked routes, compared to the shortest ones. Also, percentage of water was higher along actually walked routes (OR = 1.04, 95 % CI = 1.01–1.07). After the backward selection process, the final model for the walking route (see Table 6) showed significant differences between actual and shortest routes. There were more traffic lights, less zebra crossings and a lower percentage of sidewalks along the actual walking routes compared to the shortest routes.

**Table 4:** Characteristics of shortest walking routes compared to characteristics of actual walking routes

	Mean and ± SD or Median and interquartile range (25–75)					95 % confidence interval	
	Shortest GIS-Route		Actual GPS-Route		OR	Lower	Upper
Length of route (meter)	382.8	(270.8–579.4)	390.0	(248.8–606.7)	1.00	1.00	1.00
Land-use							
Entropy	0.40	(0.26–0.56)	0.38	(0.17–0.55)	0.11	0.00	3.78
Commercial area (%)	0.0	(0.0–0.0)	0.0	(0.0–0.0)	1.23	0.41	3.65
Residential area (%)	88.9	(82.5–95.4)	90.3	(81.8–97.5)	1.03	0.95	1.11
Recreational area (%)	2.1	(2.1–11.6)	5.3	(0.0–12.4)	1.07	0.97	1.19
Traffic area (%)	**0.0**	(**0.0–8.5**)	**0.0**	(**0.0–5.5**)	**0.76**	**0.6**	**0.96**
Residents (n per km)	77.2	57.2–94.5)	71.3	(59.3–93.5)	1.00	0.99	1.02
Aesthetics							
% Green along route	38.7	(18.3–52.9)	43.9	(19.2–62.6)	1.01	0.99	1.03
% Water along route	**12.8**	(**0.0–42.2**)	**20.1**	(**0.0–46.8**)	**1.04**	**1.01**	**1.07**
Trees (n per km)	147.5	(118.3–179.1)	156.3	(122.1–197.2)	1.00	0.99	1.01
Traffic							
Traffic lights (n per km)	0.00	(0.0–0.0)	0.00	(0.0–0.0)	1.23	0.95	1.61
Street lights (n per km)	**70.0**	**(59.4–94.8)**	**65.8**	**(50.6–84.8)**	**0.97**	**0.95**	**0.99**
Street bumps (n per km)	7.7	(1.7–14.6)	6.4	(0.0–14.9)	0.98	0.9	1.07
Accidents (n per km)	1.6	(0.0–3.3)	0.0	(0.0–2.5)	0.95	0.77	1.16
Zebra crossings (n per km)	**1.7**	(**0.0–3.6**)	**0.0**	(**0.0–2.9**)	**0.55**	**0.35**	**0.87**
Junctions (n per km)	37.8	**±**13.2	37.1	**±**16.4	1.00	0.97	1.03
% Sidewalk along route	**98.9**	(**95.5–99.9**)	**93.7**	(**77.2–100.0**)	**0.94**	**0.90**	**0.98**
Type of Street							
Main Road (%)	0.0	(0.0–16.9)	0.0	(0.0–12.7)	1.00	0.94	1.05
Residential Street (%)	44.0	**±**14.9	49.8	**±**19.0	1.04	1.01	1.08
Cycling path (%)	10.4	(10.4–26.8)	12.4	(0.0–30.2)	1.00	0.96	1.04
Pedestrian path (%)	30.0	(20.4–41.0)	24.6	(15.3–38.2)	0.93	0.89	0.98

*SD* = standard deviation, *OR* = odds ratio, *p*-values below 0.05 are printed in bold type

### Environmental characteristics of cycling routes

Results of the conditional regression analysis on differences between environmental characteristics of actually cycled routes and shortest cycling route are shown in Table 5. Similar to walking routes, cycling routes were located mostly in residential areas (80.7 %). Compared to the shortest route, a larger part of the actual cycling route travelled through recreational areas (OR = 1.06, 95 % CI = 1.01–1.11). The percentage of surface water (42.7 %) along the actual route was also significantly higher compared to the shortest route (OR = 1.03, 95 % CI = 1.01–1.05). Moreover, actually cycled routes differed from the shortest routes on all of the variables in the traffic category, i.e., more traffic lights and junctions, and less street lighting, speed bumps, accidents and zebra crossings. Most of the actual cycling routes passed through residential streets (43.9 %). This is significantly more often compared to the shortest route (OR = 1.03, 95 % CI = 1.01–1.06). Pedestrian paths were covered less on actual cycling routes compared to shortest routes (OR = 0.95, 95 % CI = 0.92–0.98).

**Table 5:** Characteristics of shortest cycling routes compared to characteristics of actual cycling routes

	Mean and ± SD or Median and interquartile range (25–75)					95 % confidence interval	
	Shortest GIS-route		Actual GPS-route		OR	Lower	Upper
Length of route (meter)	675.6	(498.8–965.3)	673.9	(459.4–1008.3)	1.00	1.00	1.00
Land-use							
Entropy	0.49	(0.33–0.69)	0.51	(0.35–0.67)	3.82	0.43	33.67
Commercial area (%)	0.00	(0.0–0.0)	0.00	(0.0–0.1)	0.88	0.71	1.09
Residential area (%)	86.5	(73.3–91.9)	84.0	(75.5–91.4)	0.98	0.94	1.02
Recreational area (%)	**9.8**	**(2.0–20.2)**	**11.9**	**(5.5–19.2)**	**1.06**	**1.01**	**1.11**
Traffic area (%)	2.0	(0.0–8.7)	1.4	(0.0–6.9)	0.90	0.82	1.00
Residents (n per km)	65.4	(45.0–84.8)	63.1	(46.5–80.7	1.00	0.98	1.01
Aesthetics							
% Green along route	49.9	(34.0–71.5)	55.7	(32.5–72.4)	1.00	0.99	1.02
% Water along route	**37.7**	**(10.2–61.3)**	**44.6**	**(15.4–68.5)**	**1.03**	**1.01**	**1.05**
Trees (n per km)	**145.6**	**(102.9–174.2)**	**140.7**	**(103.5–167.8)**	**0.99**	**0.97**	**1.00**
Traffic							
Traffic lights (n per km)	**0.00**	**(0.0–0.0)**	**0.00**	**(0.0–0.0)**	**1.29**	**1.04**	**1.58**
Street lights (n per km)	**67.7**	**±21.6**	**62.3**	**±21.0**	**0.95**	**0.93**	**0.98**
Street bumps (n per km)	**3.7**	**(1.5–9.8)**	**3.4**	**(0.8–8.3)**	**0.96**	**0.89**	**1.05**
Accidents (n per km)	**1.8**	**(0.0–3.6)**	**1.1**	**(0.0–2.9)**	**0.57**	**0.43**	**0.76**
Zebra crossings (n per km)	**1.6**	**(0.0–2.3)**	**0.8**	**(0.0–2.3)**	**0.56**	**0.39**	**0.81**
Junctions (n per km)	**33.2**	**±13.4**	**35.3**	**±12.8**	**1.03**	**1.01**	**1.06**
% Sidewalk along route	**98.7**	**(83.0–99.9)**	**93.2**	**(78.1–100.0)**	**0.96**	**0.93**	**0.99**
Type of Street							
Main Road (%)	3.4	(0.0–17.0)	7.9	(0.0–14.5)	0.99	0.95	1.02
Residential Street (%)	**40.8**	**±16.5**	**43.9**	**±15.2**	**1.03**	**1.01**	**1.06**
Cycling path (%)	22.3	(6.0–34.4)	24.1	(11.5–35.9)	1.01	0.98	1.04
Pedestrian path (%)	**22.8**	**(14.7–35.1)**	**22.0**	**(15.7–28.8)**	**0.95**	**0.92**	**0.98**

*SD* = standard deviation, *OR* = odds ratio, *p*-values below 0.05 are printed in bold type

After the backward selection process, the final model for cycling routes (see Table 6) showed differences between actual and shortest cycling routes. Compared to the shortest GIS routes, actual cycling routes had a smaller number of trees, accidents, zebra crossings, and a lower percentage of sidewalks along the route compared to the shortest cycling routes. Moreover, actually cycled routes had more traffic lights, and junctions and a higher chance of being on residential streets compared to the shortest GIS route.

**Table 6:** Final multivariate models for actual walking and cycling routes

	Beta	OR	95 % confidence interval	
			Lower	Upper
Walking				
Traffic lights	.75	2.11	1.07	4.15
Zebra crossings	−1.77	.17	.05	.58
% Residential Street	.12	1.12	1.04	1.21
% Sidewalk along route	−.09	.91	.85	.98
Cycling				
Trees	−.02	.98	.96	0.99
Traffic lights	.56	1.75	1.04	2.95
Accidents	−.56	.57	.39	.83
Zebra crossings	−1.19	.31	.14	.67
Junctions	.12	1.13	1.05	1.20
% Residential streets	.06	1.06	1.01	1.12
% Pedestrian paths	−.09	.91	.85	.98
% Sidewalk along route	−.10	.91	.85	.96

*SD* standard deviation, *OR* odds ratio

## Discussion

This cross-sectional study investigated differences in environmental characteristics of the actual walking and cycling route between home and school, compared to the environmental characteristics of the shortest route. Examining these differences provides insight in Dutch children’s route choice during active transportation between home and school.

For children that walked, median distance of the actual journey to school was 343.3 m. Actual routes for cyclists had a median distance of 673.9 m. These covered cycling distances are very similar to the average distance to a primary school in the Netherlands, which is 700 m [30]. As expected, mode of transportation between home and school was related to the distance of the route [23, 31]. The actual routes were 5.6 % and 10.9 % longer than the shortest route for walking and cycling, respectively. These detour ratios are in line with a study of Krenn et al. [14] who found that adult cyclists in Graz detoured on average 7.6 %.

In final multivariate models, there was a significant difference in the amount of zebra crossings on the actual route to school versus the shortest route. Both during their cycling and walking routes, children were less likely to cover routes with zebra crossings (walking route: OR = 0.17, 95 % CI = 0.05–0.58; cycling route: OR = 0.31, 95 % CI = 0.14–0.67). On the other hand, children did seem to use crossings with traffic lights when they were available. Possibly, children avoided walking along the busy roads when going to school, and preferably used signalized intersections to cross the main roads. Earlier, other studies have shown that such signalized intersections were associated with active travel to school [32, 33]. Most of the zebra crossings in the Netherlands are located on or near roads where speed and intensity of motorized traffic is higher. It is likely that children avoid these busy streets. This could also explain why actual routes had a lower record of accidents as measured through BRON [29] compared to the shortest route, since accidents more often occur on busier roads. Unfortunately, data on traffic intensity was only available for the arterial roads in the dataset, not for the other streets in the network and could thus not be used in the current analysis.

Moreover, on their route to school children mainly traveled through residential areas and used residential streets (49.8 % of their walking route, 43.9 % of their cycling route). This was significantly different from their shortest route to school (walking: 44.0 %, cycling:40.8 %). Typically, residential streets are spread across residential areas and have many corners, junctions and short cuts. Actual cycling routes also had significantly more junctions compared to shortest routes. It has been shown that a high connectivity is supportive of active transportation [34]. Moreover, in the Netherlands, speed and intensity of the motorized traffic is low in these residential areas. There is usually a speed limit of 30 km/h on these streets. Also, during the morning the phenomenon of ‘safety by numbers’ may play a role here. According to this theory motorists change their behavior when large numbers of cyclists or pedestrians are present [35]. Thus, these residential streets may be perceived as safe to use for cycling, despite the absence of separate bicycle paths.

Furthermore, although some studies have shown that aesthetics, or an enjoyable scenery, can be associated with active transportation [36, 37] this study did not show that actual walking or cycling routes had more visible green than shortest routes between home and school. There was no significant difference between percentage of green along the children’s actual walking and cycling routes and the percentage of green along the shortest routes on the network. On the other hand, in the unadjusted models, there was a difference in the amount of water ways visible along the actual routes, compared to the shortest routes. Both cyclists and pedestrians seemed to prefer routes that had a higher percentage of visible surface water along the route. This could be due to aesthetics of the route [36, 37], but another explanation could be that routes along the water are generally more safe because of the buffer that water ways offer from other traffic.

Contrary to what would be expected, the percentage of sidewalks along the actual walking route was lower than the percentage of sidewalks along the shortest route. Previous studies have shown that sidewalk presence is positively associated with walking to school [32]. In the current study, large parts of the walking routes (87 %) were located on or near a sidewalk, but not significantly different compared to the percentage of sidewalks along the shortest routes. Thus, these results may be explained by the incompleteness of the pedestrian street network, where still not every possible path could be mapped.

### Strengths & limitations

This study used a novel approach to investigate environmental characteristics of active transportation to school. In contrast to previous studies, analysis of walking and cycling routes were separated as both transportation modes require different street infrastructure. The current study investigated environmental characteristics of the actually traveled route by investigating environmental correlates within a range of 25 m of the GPS signal. By using this relatively small buffer the actual exposure to the environment was much better represented than when using large circular buffers (e.g., 400 m) around the house that are commonly used to represent environmental exposure during active transportation. It can be argued that people are not influenced by all of the features that are encompassed by these large circular buffers, e.g., because they never interact with these distant features [21]. Still, as buffers are used in the current study, the modifiable area unit problem may have played a role in this analysis as well. The modifiable area unit problem represents a phenomenon in geospatial analysis where observed aggregated values are different dependent on the boundaries that are drawn [32], in this case buffer size. This study used a 25 m buffer to optimally discriminate between the GIS derived route and the GPS route. Larger buffers that are also commonly used, i.e., 100 m or 250 m, make it hard to find differences between the two routes because of the increasing overlap of the buffers. Moreover, two other comparable studies also used a similar buffer size of 25 m [14, 38]. Another study used buffers of 100 m to identify food outlets and physical activity facilities, but used a similar buffer of 20 m to join the GPS points to the road network [13].

Also, despite only using the shortest walking and cycling GPS route in the analysis, multi-destination tracks were still present in our sample. Moreover, the recorded GPS tracks were not matched to the street network during the data handling process. Instead, the recorded GPS routes were buffered. So, in the analysis the buffered ‘raw’ GPS signal of the actual route was compared with the buffered street network. This was done to resemble the actually walked or cycled routes as closely as possible. Sidewalks, for example, are generally aligned perfectly aside the buffered street network, whereas the actual GPS signal follows a more arbitrary path that can deviate from the street network. Thus, part of the sidewalks along the route are missed. This was partially solved by using a buffer, in this case 25 m, which also compensated for the inaccuracy in the GPS signal. Schipperijn et al. [39] showed that with the BT Q1000XT-model of the GPS, median error for walking trips was 3.9 m, and for cycling trips 2.0 m, but still >20 % of GPS points fell outside of 10 m of the expected location. These methodological constraints may have influenced results of this study, underestimating the presence of certain characteristics along the actual active transportation routes.

Data collection took place in the more suburban parts of the Netherlands. Children who travel in more urban or more rural areas may use different routes to school. Although a similar analysis for rural routes would be less interesting because of the lack of alternative routes, it would be interesting to see if active transportation routes in the bigger cities are similar. Furthermore, most of the data collection took place around spring and the beginning of summer. This could have influenced transportation behavior of the children. It is well known that seasonal changes, e.g., hours of daylight, weather, can have a large impact on daily physical activity levels and mode of transportation [40]. In the winter when it is still dark during morning trips, for example street lighting may play a more significant role in route choice for walking and/or cycling. Thus, some of the results of the current study may be the consequence of methodological choices and challenges, e.g., cross-sectional study, size of the buffers, aligning GPS signals with the pedestrian street network, multi-destination trips.

## Conclusion

In the current study, actual cycling routes had significantly more traffic lights and a higher connectivity than shortest routes. Moreover, children that cycled to school avoided streets with a high incidence of accidents. Both on their walking and cycling routes, children seemed to prefer residential streets over other type of streets, but avoided streets with zebra crossings. Most of the differences between actual and shortest routes may be explained by the preference of children (and their parents) to avoid walking or cycling along the busy roads on their way to school. Thus, this study seems to confirm the importance of traffic safety for active transportation to school.

## Additional file

Additional file 1:
**Table S1**. Description of the GIS-variables used in the comparison of the shortest and actual route. **Table S2**. Descriptive statistics of GPS tracks (*N* = 1,249) between home and school of 184 children. (DOCX 23 kb)

## Abbreviations

ATS: active transportation to school
GPS: global positioning system
GIS: geographic information system
CBS: Centraal Bureau Statistiek (Statistics Netherlands)
