# Supplementary material for: Children’s route choice during active transportation to school: difference between shortest and actual route
Source: Int J Behav Nutr Phys Act. 2016 Apr 12;13:48. doi: 10.1186/s12966-016-0373-y (PMC4830076; doi:10.1186/s12966-016-0373-y)
Supplement: Additional file 1: — Table S1. Description of the GIS-variables used in the comparison of the shortest and actual route. Table S2. Descriptive statistics of GPS tracks (N = 1,249) between home and school of 184 children. (DOCX 23 kb) [file 12966_2016_373_MOESM1_ESM.docx]

Table S1. Description of the GIS-variables used in the comparison of the shortest and actual route

| **Variable** | **Source** | **Type of GIS-data*** | **Explanation** |
| --- | --- | --- | --- |
| **Street Network** | Top10NL, OpenStreetMap (OSM), satellite images | Polylines | Constructed using the road centerlines available in the TOP10NL database (topographic map of the Netherlands, scale 1: 10.000 provided by the Dutch Land use register Kadaster). To complete the street network, centerlines of missing streets were manually added based on Open Street Map (OSM) data and satellite images from LuchtfotoNL (2014). |
| Shortest route length (meter) | Street Network | Polylines | Shortest routes were calculated with the Network Analyst tool in ArcGis 10.2 and were based on the shortest routes on the street network between the x,y-location of the home and school building. |
| Actual route length (meter) | GPS-tracks | Polylines | Locations of the home address and school building of the children were determined based on clusters in the GPS data. Next, each GPS track between the home address and the school building was identified with an automatic procedure. Trips going in both directions (i.e. home or school) were eligible to be included in the analysis. |
| **Land-use** |  |  |  |
| Entropy | CBS Land use data | Polygons | A four-category entropy index in which 0 stands for no diversity, while 1 means that there is an equal distribution of land use. A distinction was made between the following entropy categories: commercial areas, residential areas, recreational areas, and transport areas. |
| % Commercial area | CBS Land use data | Polygons | Public facilities, office space, industrial, retail, or construction areas as classified by the CBS Land use data. Reported as a percentage of the total surface area of the route. |
| % Residential area | CBS Land use data | Polygons | Areas classified as residential areas in the CBS land use data. Reported as a percentage of the total surface area of the route. |
| % Recreational area | CBS Land use data | Polygons | Areas classified as green, playgrounds, parks, water and sports facilities in the CBS Land use data. Reported as a percentage of the total surface area of the route. |
| % Transport area | CBS Land use data | Polygons | Areas classified as main roads, railways or airports in the CBS Land use data. Reported as a percentage of the total surface area of the route. |
| Residents  (n per km) | CBS Squares | Polygons | CBS squares, derived from the Dutch Statistics Center, provided number of residents in 100 by 100 meter cells. Surface area within a 25 meter of the route . Reported as estimated number of residents per kilometer of route. |
| **Aesthetics** |  |  |  |
| % Green along route | TOP10NL | Polygons | Calculated based on the areas of green from TOP10NL, these surface areas represent neighborhood green spaces (e.g. bushes, grass plots, woods). Areas of green were buffered with 25 meter. Length of the route within these buffers where divided by the total route length and reported as a percentage of the route. |
| % Water along route | TOP10NL | Polygons | Calculated based on the surface areas of water from TOP10NL, these surface areas represent natural water ways (e.g. ponds, rivers, lakes). Areas of water were buffered with 25 meter. Length of the route within these buffers where divided by the total route length and reported as a percentage of the route. |
| Trees  (n per km) | local land use registry data | Points | Number of trees within the buffer of 25 meter of the route. Reported as the number of points per km of route. |
| **Traffic** |  |  |  |
| Traffic lights  (n per km) | local land use registry data, OSM | Points | Number of traffic lights within the buffer of 25 meter of the route. Missing traffic lights were manually added based on Open Street Map data. Reported as the number of points per km of route. |
| Street lights  (n per km) | local land use registry data | Points | Number of lampposts within the buffer of 25 meter of the route. Reported as the number of points per km of route. |
| Street bumps  (n per km) | local land use registry data, OSM, satellite images | Points | Number of street bumps within the buffer of 25 meter of the route. Missing street bumps were manually added based on Open Street Map data and satellite images from LuchtfotoNL. Reported as the number of points per km of route. |
| Accidents  (n per km) | BRON | Points | Number of traffic related accidents recorded in a national database through official reports or registration sets from the police. |
| Zebra crossings (n per km) | local land use registry data, OSM, satellite images | Points | Number of zebra crossings within the buffer of 25 meter of the route. Missing zebra crossings were manually added based on Open Street Map data and satellite images from LuchtfotoNL. Reported as the number of points per km of route. |
| Junctions  (n per km) | Street network | Points | Number of junctions within the buffer of 25 meter of the route. Reported as the number of points per km of route. |
| % Sidewalk along route | local land use registry data | Polygons | Calculated based on the data on sidewalks provided by the local municipality. Sidewalk areas were buffered with 25 meter. Length of the route within these buffers where divided by the total route length and reported as a percentage of the route. |
| **Type of Street** |  |  |  |
| %Main Road | Street Network | Polylines | All arterial roads, including highways within a buffer of 25 meter of the route. Speed limits on these roads are typically 50 km/h or higher. When cyclists travel along these roads, they are directed to a separately marked cycling lane. Pedestrians use the sidewalk when these are available. Reported as a percentage of total length of roads. |
| %Residential Street | Street Network | Polylines | Roads going through residential areas. Residential streets are used for all modes of transportation, thus motorized traffic shares the streets with cyclists while pedestrians are directed to the sidewalk. Maximum speed of motorized traffic on residential streets is usually low, with a maximum of 30 km/h. Reported as a percentage of total length of roads. |
| %Cycling path | Street Network | Polylines | Separate cycling paths designed for use by cyclists. Bike lanes marked by a line on the main road were not included in this category. Reported as a percentage of total length of roads. |
| %Pedestrian path | Street Network | Polylines | Separate paths designed for use by pedestrians, e.g., pathways through parks or other recreational facilities, shortcuts between houses. Reported as a percentage of total length of roads. |

* Type of GIS-data: a) Points: represented by a x,y -coordinate b) Polylines: a sequence of points defining a connected series of line segments c) Polygons: a closed chain of line segments forming a plane.

Table S2. Descriptive statistics of GPS tracks (N=1,249) between home and school of 184 children.

| **Mode of transport** |  | **Mean** | **Standard Deviation** | **Median** | **25^th^ percentile** | **75^th^ percentile** |
| --- | --- | --- | --- | --- | --- | --- |
| ***Walking*** | Distance (meters) | 405.3 | 310.9 | 343.3 | 213.0 | 549.8 |
| *N =211* | Duration (minutes) | 7.7 | 10.4 | 5.3 | 3.0 | 8.8 |
| *n = 67* | Average speed (km/h) | 3.7 | 1.2 | 3.8 | 3.0 | 4.5 |
|  | Max Speed (km/h) | 10.5 | 7.9 | 9.2 | 6.8 | 11.2 |
|  |  |  |  |  |  |  |
| ***Cycling*** | Distance (meters) | 1066.3 | 1142.1 | 817.4 | 563.6 | 1186.0 |
| *N = 831* | Duration (minutes) | 14.1 | 29.7 | 6.3 | 4.1 | 10.5 |
| *n =162* | Average speed (km/h) | 7.7 | 3.5 | 7.4 | 4.9 | 10.4 |
|  | Max Speed (km/h) | 19.7 | 24.0 | 18.3 | 15.9 | 20.8 |
|  |  |  |  |  |  |  |
| ***Motorized transport*** | Distance (meters) | 4900.0 | 6734.9 | 2082.0 | 1076.8 | 5770.3 |
|  | Duration (minutes) | 75.3 | 199.8 | 8.2 | 4.9 | 53.5 |
| *N = 207* | Average speed (km/h) | 12.6 | 7.3 | 12.6 | 6.7 | 17.4 |
| *n = 70* | Max Speed (km/h) | 56.8 | 30.5 | 52.2 | 40.1 | 63.2 |

N= number of tracks recorded for each transportation mode, n=number of children
